# HMGA1 Regulates the Expression of Replication-Dependent Histone Genes and Cell-Cycle in Breast Cancer Cells

**DOI:** 10.3390/ijms24010594

**Published:** 2022-12-29

**Authors:** Sara Petrosino, Sabrina Pacor, Silvia Pegoraro, Virginia Anna Gazziero, Giulia Canarutto, Silvano Piazza, Guidalberto Manfioletti, Riccardo Sgarra

**Affiliations:** 1 Department of Life Sciences, University of Trieste, 34127 Trieste, Italy; 2 International Centre for Genetic Engineering and Biotechnology (ICGEB), 34149 Trieste, Italy

**Keywords:** TNBC, HMGA1, RD-HIST, NPAT, cell-cycle, epirubicin

## Abstract

Breast cancer (BC) is the primary cause of cancer mortality in women and the triple-negative breast cancer (TNBC) is the most aggressive subtype characterized by poor differentiation and high proliferative properties. High mobility group A1 (HMGA1) is an oncogenic factor involved in the onset and progression of the neoplastic transformation in BC. Here, we unraveled that the replication-dependent-histone (RD-HIST) gene expression is enriched in BC tissues and correlates with HMGA1 expression. We explored the role of HMGA1 in modulating the RD-HIST genes expression in TNBC cells and show that MDA-MB-231 cells, depleted of HMGA1, express low levels of core histones. We show that HMGA1 participates in the activation of the *HIST1H4H* promoter and that it interacts with the nuclear protein of the ataxia-telangiectasia mutated locus (NPAT), the coordinator of the transcription of the RD-HIST genes. Moreover, we demonstrate that HMGA1 silencing increases the percentage of cells in G0/G1 phase both in TNBC and epirubicin resistant TNBC cells. Moreover, HMGA1 silencing causes an increase in epirubicin IC_50_ both in parental and epirubicin resistant cells thus suggesting that targeting HMGA1 could affect the efficacy of epirubicin treatment.

## 1. Introduction

Breast cancer (BC) is a world health problem and the major cause of cancer death in women [1]. BC stands out for its heterogeneity in pathological features, molecular signatures, and clinical outcomes [2]. It is divided into five intrinsic subtypes including luminal A, luminal B, HER2-overexpressing, basal-like, and normal-like tumors [3]. Histologically, basal-like subtype is usually high grade and with high proliferation rate. Moreover, this subtype generally displays a negative immunophenotype for estrogen receptor (ER), progesterone receptor (PR), and epidermal growth factor receptor 2 (HER2). For this reason, basal-like tumors are also referred to as triple negative breast cancer (TNBC). TNBC represents 10%–15% of all BC and shows a highly aggressive phenotype [3,4]. The patients affected by TNBC often develop distant metastasis leading to a poor prognosis [3]. TNBC is mainly treated with primary chemotherapy and adjuvant treatments are generally recommended [4]. The most employed adjuvants are anthracyclines, such as epirubicin, that in combination with cyclophosphamide and taxanes confer survival advantage to the patients. As well, paclitaxel-plus-carboplatin regimen can be an effective alternative too [5,6].

The architectural transcription factor HMGA1 is a key oncogenic protein in BC [5]. HMGA1 is characterized by the presence of three AT-hook domains that confer the ability to bind AT-rich DNA sequences. HMGA1 establishes interactions both with DNA and several transcription factors and co-activators, facilitating the binding of the latter onto DNA promoter/enhancer regions thus regulating the transcription of several genes [6]. HMGA1 is overexpressed in BC upon dysregulation of different transcriptional and post-transcriptional mechanisms [5]. BC tissues express high levels of HMGA1, and this could have a prognostic value [7,8,9,10,11,12]. Pioneering work demonstrated that the overexpression of HMGA1 in BC cells leads to the acquisition of aggressiveness and metastatic traits [13]. Moreover, interfering with its expression causes a reversion of the cancer phenotype [14]. Recently the role of HMGA1 expression in BC cells has been thoroughly investigated showing that HMGA1 influences the expression of a plethora of factors inserted in different pathways involved in cancer onset and development. These pathways include stemness, epithelial-to-mesenchymal-transition (EMT), angiogenesis, and DNA repair [15,16,17,18,19].

The nucleosome is formed by DNA wrapped around a proteinaceous core made by the close association of the four core histones (H2A, H2B, H3, and H4). Nucleosomes are assembled in high-ordered structures by histone H1 that binds DNA at the entry/exit sites of nucleosomes. DNA replication and its subsequent packaging into high-ordered structures is a regulated process that needs tight synchronization with the expression of the replication-dependent histones (RD-HISTs). The RD-HISTs mRNA increase 35-fold as cells enter S-phase, when there is a high increase in DNA content and the necessity to provide a structural scaffold [20]. Notwithstanding the fact that each histone gene has its own specific promoter, the coordinated expression of the RD-HIST genes is guaranteed by their clustering in the genome. In humans there are three main clusters: histone cluster 1 (HIST1), the largest cluster, located on chromosome 6 (6p21–p22) and two smaller clusters, HIST2 and HIST3, are present on chromosome 1 (1q21 and 1q42, respectively). All the histone H1 genes are in the HIST1 cluster. HIST1 cluster contains six histone H1 genes, 12 H2A genes, 15 H2B genes, 10 H3 genes, and 12 H4 genes; HIST2 contains 3 H2A genes, 1 H2B gene, 1 H3 gene, and 1 H4 gene; HIST3 contains 1 H2A gene, 1 H2B gene, and 1 H3 gene [21]. The regulation of transcription of the RD-HIST genes is provided by the nuclear protein of the ataxia-telangiectasia mutated locus (NPAT) and other proteins that establish the histone locus body (HLB), a nuclear proteinaceous membrane less organelle (PMLO) that is the site of recruitment for all the factors necessary for the transcription and processing of the histone clusters [22]. Another step of regulation of RD-HISTs mRNA expression is provided by the binding of stem-loop binding protein (SLBP) to the 3’ end of RD-HISTs mRNA. The SLBP is required for all steps of histone mRNA metabolism: stabilization, translation, and degradation [20,23,24]. Since SLBP participates in histone mRNA processing during their entire life, the amount of histone mRNAs is also linked to the amount of SLBP. Indeed, the regulation of SLBP expression level during the cell-cycle is among those mechanisms by which the cell-cycle regulates the mammalian histone mRNAs levels [25].

In this work we provide evidence that HMGA1 is involved in the modulation of the expression of RD-HIST genes and that this can have an impact on the cell-cycle distribution of BC cells. This experimental evidence provides an additional mechanism through which HMGA1 could exert its oncogenic activity.

## 2. Results

### 2.1. HMGA1 Modulates the Expression of RD-HIST Genes in BC Cells

**Figure 1:**
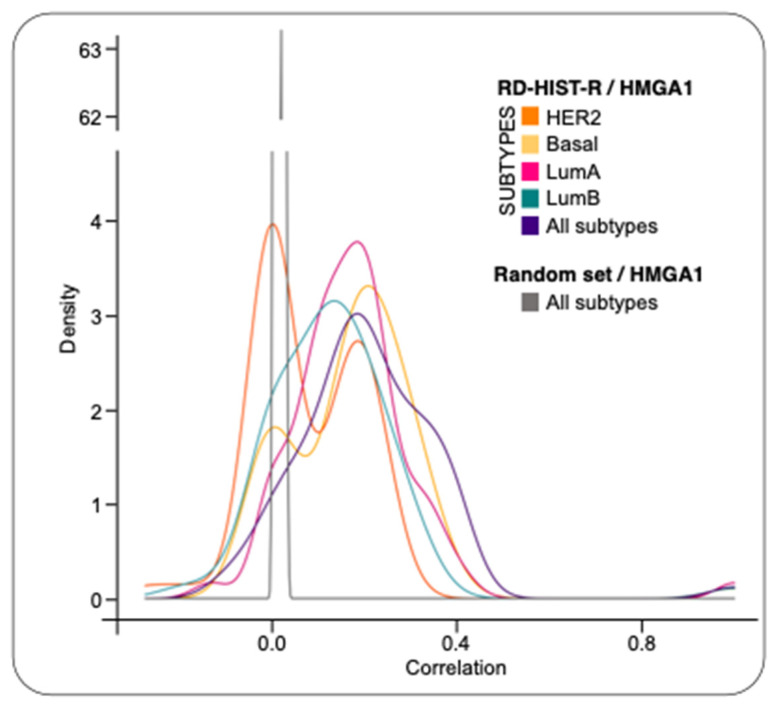
Correlation between HMGA1 and RD-HIST-R genes on different BC subtypes. Distributions of correlation values between HMGA1 and RD-HIST-R genes in each subtype, compared to randomly selected non-histones genes as control. ECDFs were generated for each subtype and their corresponding control random sets.

We performed a differential expression analysis, comparing BC expression profiles to normal samples, to investigate the expression of RD-HIST genes in BC and its connection with HMGA1. We compared the RNA-seq data, generated from more than three thousand independent tumors deriving from the prospective population-based multicenter SCAN-B study, and the GTEx dataset of healthy breast mammary tissue. Results showed that most of the RD-HIST genes (58 out of 63—RD-HIST-R genes) and HMGA1 were differentially expressed in tumors as well as across different BC subtypes (Supplementary Table S1). As a second step, we computed the correlations between HMGA1 and RD-HIST-R genes for all samples and for each subtype. Moreover, we plotted the distributions of correlations together with a thousand gene-sets composed of random genes. The RD-HIST-R gene set showed a moderate to medium correlation of expression respect to HMGA1 in all subtypes and in single subsets other than the HER2 subtype (Figure 1). Notably, to have a statistical support, we created an empirical cumulative distribution functions (ECDFs) for each subtype and their corresponding control random sets and compared them with the Kolmogorov-Smirnov test (see Supplementary Figure S1). These correlation analyses suggested an intriguing hypothesis that HMGA1 could be a modulator of RD-HIST genes expression. 

**Figure 2:**
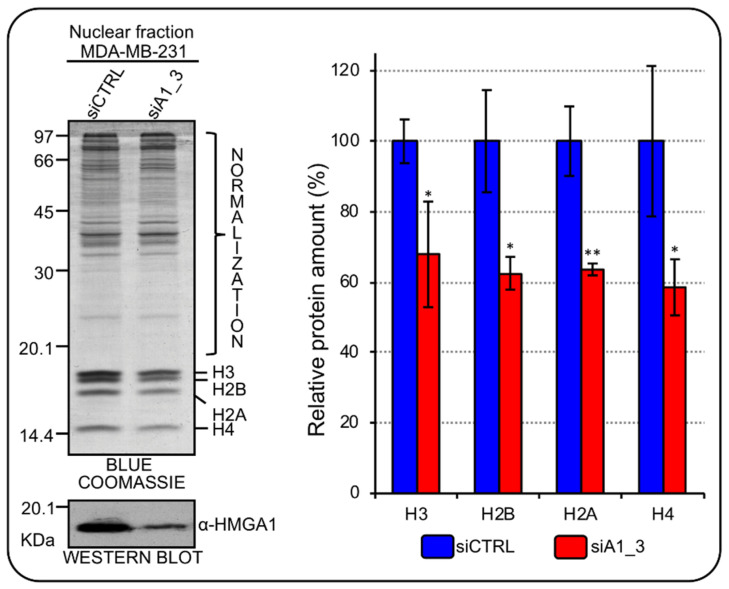
HMGA1 regulates core histones protein expression in MDA-MB-231 cells. Blue Coomassie stained gel was used to assess core histones protein amount in the nuclear fraction of MDA–MB–231 cells silenced for HMGA1 (siA1_3) and with control siRNA (siCTRL). The upper part of the gel (not including histones bands) was exploited for total protein normalization. The western blot below the Blue Coomassie staining confirms the silencing of HMGA1. Histogram graphs represent densitometric analyses (siCTRL versus siA1_3) of protein staining. The upper part of the stained gel was used to normalize the total amount of core histones. Bars indicate the mean. Standard deviations are shown (*n* = 4). Statistical significance was assessed with Student’s *t* test (*: *p* ≤ 0.05; **: *p* ≤ 0.01).

HMGA1 gene is significantly overexpressed in BC subtypes with respect to normal tissues [15] and its down regulation, in different TNBC cell lines, negatively impacts on cell proliferation [16]. Therefore, given the tight connection between cell-cycle and RD-HIST genes expression [21], we evaluated whether HMGA1 could be involved in the modulation of histone protein expression levels by analyzing the global histone content in HMGA1 depleted MDA-MB-231 cells. Following HMGA1-silencing, cells underwent a reproducible and evident mesenchymal-to-epithelial transition, changing from a spindle-like fibroblastic phenotype towards a flattened and polygonal morphology, as previously described [15]. We then extracted nuclear proteins from MDA-MB-231, silenced (siA1_3) or not (siCTRL) for HMGA1, and performed SDS-PAGE analysis with Blue Coomassie staining to assess whole core histones protein amount since histones are easily detectable in the lower zone (between 20 and 10 kDa) of SDS-PAGE separations. In HMGA1-depleted MDA-MB-231 cells there was a significant decrease in all core histone protein levels. These results were also confirmed in a second TNBC cell line (HBL-100) (Figure 2; Supplementary Figure S2).

**Figure 3:**
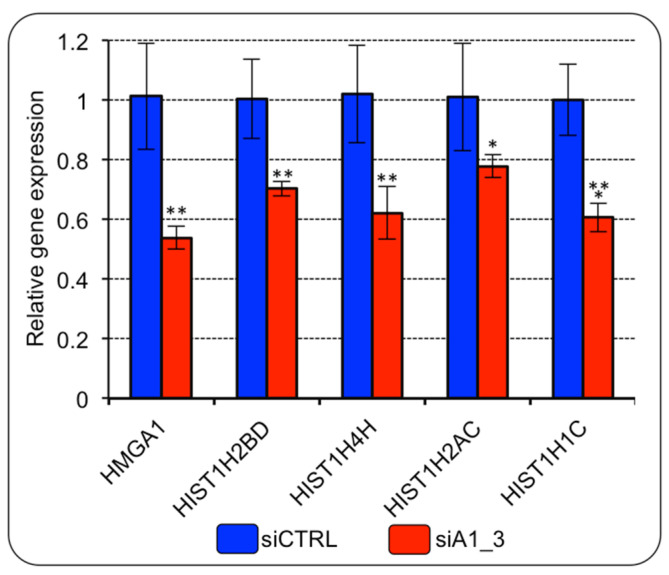
HMGA1 regulates the expression of histone genes belonging to HIST1 in MDA-MB-231 cells. RT-qPCR gene expression analyses of four genes of the RD-HIST module in MDA–MB–231 cells silenced for HMGA1 (siA1_3) or treated with control siRNA (siCTRL). The levels of HMGA1 expression are included in the panel as control for silencing efficiency. Bars indicate the mean. Standard deviations are shown (*n* = 4). Statistical significance was assessed with Student’s *t* test (*: *p* ≤ 0.05; **: *p* ≤ 0.01; ***: *p* ≤ 0.001).

To further verify whether HMGA1 expression could modulate histone gene expression in BC cells, we evaluated the expression level of six histone genes among those that turned out to be over-expressed in BC tissues (i.e., HIST1H2BD, HIST1H4H, HIST1H2AC, HIST1H1C, HIST1H2BG, and HIST1H2BK) (Supplementary Table S1). HMGA1 silencing led to the down regulation of the expression of all the histone genes evaluated (Figure 3). HIST1H2BG and HIST1H2BK were not included since they were not detectable with our RT-qPCR analysis.

### 2.2. HMGA1 Activates the HIST1H4H Promoter

**Figure 4:**
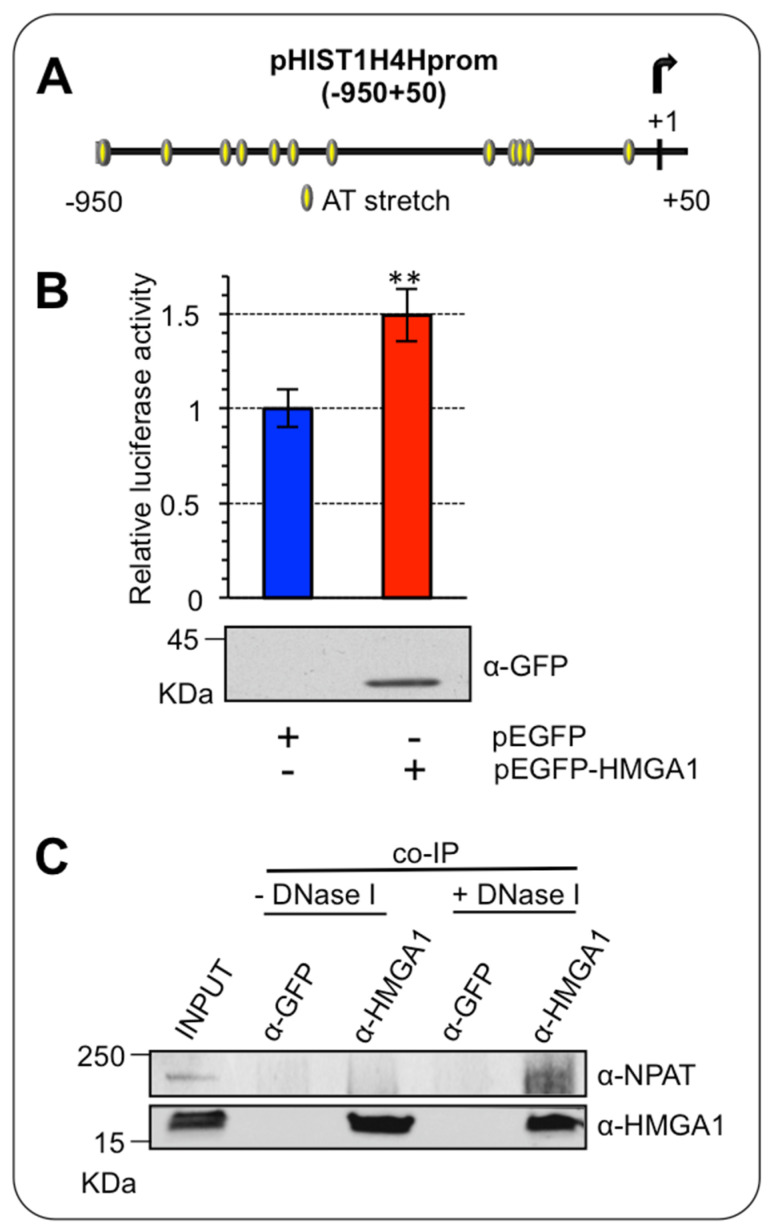
HMGA1 activates HIST1H4H/H4h promoter. (**A**) Schematic representation of the putative HMGA1-binding sites (from −950 to +50) on the promoter region of HIST1H4H. (**B**) HEK293T cells were transiently co-transfected with the luciferase reporter plasmid pGL4.11-H4H and the expression plasmid pEGFP-HMGA1 or pEGFP empty vector as control. pRL-CMV Renilla luciferase expression vector was included to normalize for transfection efficiency. Values are reported as relative luciferase activity compared to cells transfected with the pEGFP empty vector. The data are represented as means. Standard deviations are shown (*n* = 3). Statistical significance was assessed with Student’s *t* test (**: *p* ≤ 0.01). At the bottom is shown the western blot of total cell lysates to check for GFP-HMGA1 expression, using an α-GFP antibody. (**C**) Co-immunoprecipitation (co-IP) of HMGA1 and NPAT. The experiment was performed with either the negative control (α-GFP) or the α-HMGA1 antibody on MDA-MB-231 cell lysates in presence or absence of DNase I. The input and the immunoprecipitated proteins were subjected to western blot analysis with the α-HMGA1 and the α-NPAT antibodies.

We wondered whether HMGA1 could have a direct role in the modulation of RD-HIST gene expression. Thus, we tested whether HMGA1 transcriptionally regulates *HIST1H4H,* which, according to our analyses, was overexpressed in BC and was down regulated upon HMGA1 silencing. The promoter region of *HIST1H4H* contains several AT-rich DNA stretches that constitute putative HMGA1-binding sites (Figure 4A). We performed a luciferase assay overexpressing a GFP-tagged form of HMGA1 (pEGFP-HMGA1) together with a Luciferase reporter gene construct (pGL4.11-H4H) containing the promoter region, from – 950 to + 50, of *HIST1H4H* in HEK293T cells. Overexpression of HMGA1 increased the activity of the promoter, indicating that HMGA1 could potentially induce *HIST1H4H* expression in vivo (Figure 4B).

HMGA1 is an architectural chromatin factor, involved in the modulation/facilitation of the activity of the transcription factors via direct protein-protein interaction [26]. Therefore, we investigated whether HMGA1 could modulate histone expression by physically interacting with the major modulator of histone transcriptional regulation, the co-activator NPAT [22,27]. To this end, we immunoprecipitated the endogenous HMGA1 protein in MDA-MB-231 cells, which express high levels of HMGA1 and looked at its association with NPAT. HMGA1 can bind both DNA and nuclear factors, at the same time, being involved in DNA/protein macromolecular complexes [28]. We thus performed immunoprecipitation experiments in two different conditions, either in the presence or absence of DNA, to increase the probability of detecting a protein/protein interaction that could be hidden by the formation of large DNA-bound macromolecular complexes. We showed a NPAT/HMGA1 interaction uniquely in the absence of DNA, after DNaseI treatment (Figure 4C). This result is consistent with the ability of NPAT to exert its effects on the transcription of RD-HIST genes by interacting with different factors without directly binding to DNA [27], suggesting that the presence of DNA may promote a tight HMGA1/NPAT/DNA complex that could mask HMGA1 to the antibody used for the immunoprecipitation.

**Figure 5:**
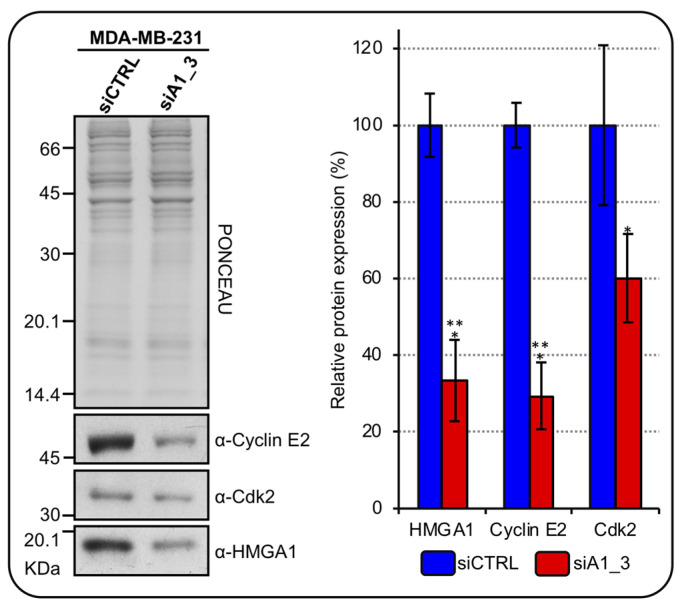
HMGA1 regulates cyclin E2 and Cdk2 expressions in MDA-MB-231 cells. Western blot analyses assess cyclinE2 and Cdk2 protein expression in MDA–MB–231 cells silenced for HMGA1 (siA1_3) or treated with control siRNA (siCTRL). Representative WB analyses, on the left, are shown together with red ponceau stained membrane to verify total protein normalization. The histogram graph on the right refers to densitometric analysis of western blot (siCTRL and siA1_3). Bars indicate the mean. Standard deviations are shown (*n* = 3). Statistical significance was assessed with Student’s *t*-test (*: *p* ≤ 0.05; ***: *p* ≤ 0.001).

Cyclin E2 interacts and colocalizes with NPAT at the level of HLB. Moreover, its expression in BC correlates with the expression of RD-HIST [29] and it also mediates the Cdk2 phosphorylation-dependent activation of NPAT [30]. Since we previously showed that HMGA1 regulates the transcription of CCNE2 [31], we tested whether HMGA1 can regulate CDK2 expression as well. Silencing HMGA1 in MDA-MB-231 cells down regulated the expression of both cyclin E2 and Cdk2 (Figure 5). 

**Figure 6:**
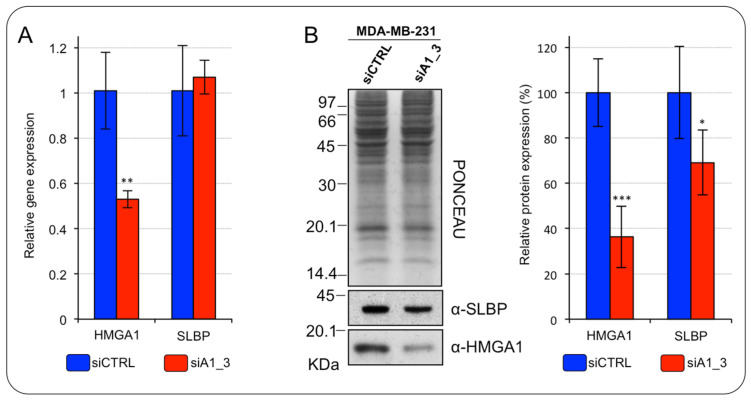
HMGA1 regulates the SLBP expression level in MDA-MB-231 cells. (**A**) RT-qPCR analyses of HMGA1 and SLBP in MDA–MB–231 cells silenced for HMGA1 (siA1_3) or treated with control siRNA (siCTRL). Bars indicate the mean. Standard deviations are shown (*n* = 4). Statistical significance was assessed with Student’s *t*-test (*: *p* ≤ 0.05; **: *p* ≤ 0.01; ***: *p* ≤ 0.001). (**B**) Western blot analyses of HMGA1 and SLBP protein expression in MDA–MB–231 cells silenced for HMGA1 (siA1_3) or treated with control siRNA (siCTRL). Representative WB analyses are shown together with red ponceau stained membrane as normalization. The histogram graph on the right refers to densitometric analysis of western blot (siCTRL versus siA1_3). Bars indicate the mean. Standard deviations are shown (*n* = 4). Statistical significance was assessed with Student’s *t*-test (*: *p* ≤ 0.05; **: *p* ≤ 0.01; ***: *p* ≤ 0.001).

At post-transcriptional level, the expression of histone mRNAs is mainly controlled by the SLBP protein. Indeed, SLBP stabilizes histone mRNAs and ensures their translation allowing the coordination between S-phase entry and the S-phase expression of histones [20]. For this reason, the SLBP is subjected to a tight cell-cycle dependent regulation at the translational level. Consequently, we checked if SLBP expression level is modulated in HMGA1-depleted MDA-MB-231 cells. Although no significant changes of the SLBP mRNA level were observed (Figure 6A), the SLBP protein level was significantly, albeit modestly, down regulated in HMGA1-depleted MDA-MB-231 cells (Figure 6B). 

The results obtained so far were coherent with our hypothesis and suggest that HMGA1 could play a role in regulating, directly and/or indirectly, RD-HIST genes expression, both at transcriptional and post-transcriptional level. 

### 2.3. HMGA1 Silencing Impacts Cell-Cycle Progression in MDA-MB-231 Cells

**Figure 7:**
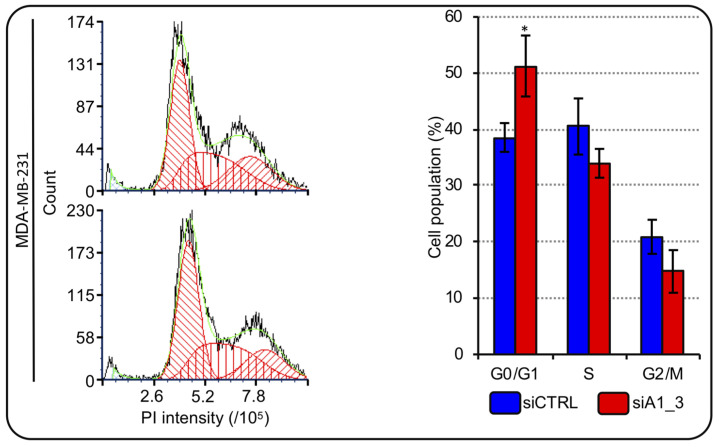
HMGA1 depletion influences MDA-MB-231 cell-cycle progression. Representative graphs showing the staining with propidium iodide (PI) of cells silenced for HMGA1 (siA1_3), lower left part, or treated with control siRNA (siCTRL), upper left part. The histogram graph on the right shows the relative percentage count of MDA-MB-231 cells in G0/G1-, S- and G2/M-phase of cell-cycle for both conditions (siCTRL and siA1_3). Bars indicate the mean. Standard deviations are shown (*n* = 3). Statistical significance was assessed with Student’s *t*-test (*: *p* ≤ 0.05).

We showed that HMGA1 is involved in the expression modulation of RD-HIST genes; therefore, we investigated the possibility that HMGA1 modulates cell-cycle in TNBC cells. We analyzed the cell-cycle distribution of MDA-MB-231 cells after HMGA1 silencing. We observed that, after 72 hours, the silencing of HMGA1 resulted in a significant increase in the number of MDA-MB-231 cells in the G0/G1 and a decrease in the number of cells in S-phase and in G2/M (Figure 7). Employing a second TNBC cell line – HBL-100 - we highlighted that the silencing of HMGA1 significantly decreased the abundance of cells in S-phase and increased cells in G2/M phase (Supplementary Figure S3). These data showed that HMGA1 expression is important for cell-cycle progression and perturbations of its expression levels cause alterations that suggest a decrease in the proliferation rate (increased G0/G1- and decreased S-phase cell population distribution).

### 2.4. HMGA1 Silencing Impacts Cell-Cycle G1-S Phase Progression in Epirubicin Resistant (EpR) MDA-MB-231 Cells

**Figure 8:**
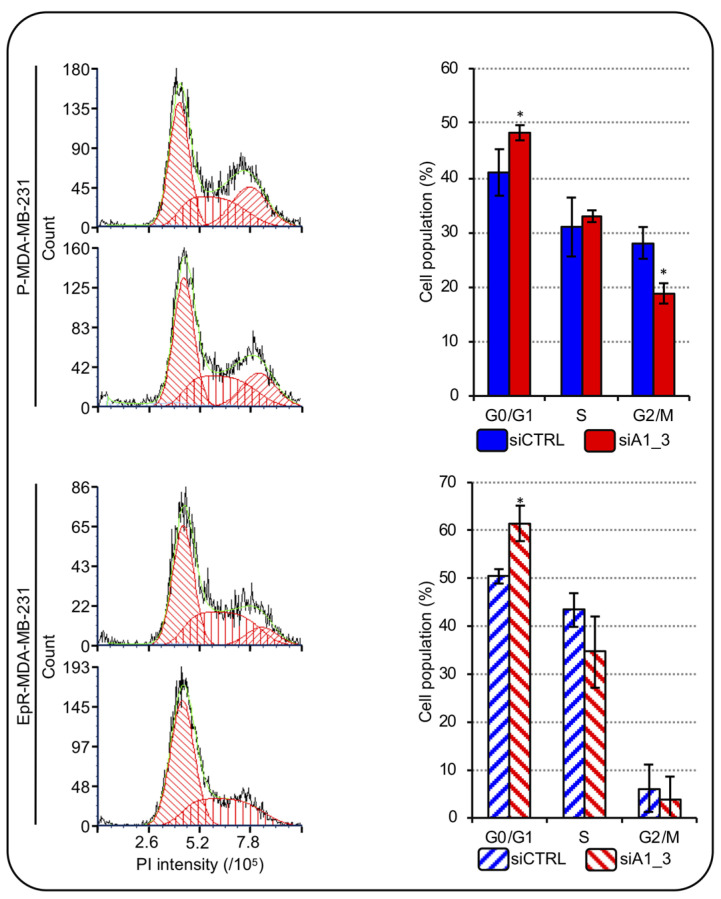
HMGA1 depletion influences EpR-MDA-MB-231 cell-cycle progression. Representative images showing the staining with PI of P-MDA-MB-231 and EpR-MDA-MB-231 cells both silenced for HMGA1 (siA1_3) or for no-target with the control siRNA (siCTRL) (left part). The histogram graphs refer to the relative percentage count of P-MDA-MB-231 and EpR-MDA-MB-231 cells in G1-, S- and G2-M-phase of cell-cycle for both conditions (siCTRL and siA1_3) analyzed (right part). Bars indicate the mean. Standard deviations are shown (*n* = 3). Statistical significance was assessed with Student’s *t*-test (*: *p ≤* 0.05).

The most employed adjuvants in TNBC treatment are anthracyclines and drug resistance development represent a frequent problematic issue. Epirubicin is the most utilized anthracycline [4] and the possibility to counteract the effects of HMGA1 in combination with drugs has been suggested as a possibility to be exploited to target TNBC [32]. We wondered whether silencing HMGA1 could modulate the sensitivity of TNBC cells to epirubicin treatment. To this end, we developed MDA-MB-231 cells resistant to epirubicin (EpR-MDA-MB-231) (Supplementary Figure S4) and evaluated the cell-cycle distribution of EpR-MDA-MB-231 and parental-MDA-MB-231 (P-MDA-MB-231) cells depleted or not of HMGA1. The silencing of HMGA1 in P-MDA-MB-231 cells resulted in a significant, albeit modest, increase in the number of cells in the G0/G1-phase accompanied by a significant decrease in the number of cells in G2/M-phase (Figure 8). These data agreed with the ones obtained in MDA-MB-231 cells (Figure 7). In addition, we observed that the silencing of HMGA1 in EpR-MDA-MB-231 cells resulted in a significant increase in the number of cells in the G0/G1-phase and a decrease in the number of cells in S-phase and G2/M-phase (Figure 8). Therefore, depletion of HMGA1 in both EpR-MDA-MB-231 and P-MDA-MB-231 cells resulted in a significant alteration of cell-cycle distribution increasing the number of cells in G0/G1-phase.

**Figure 9:**
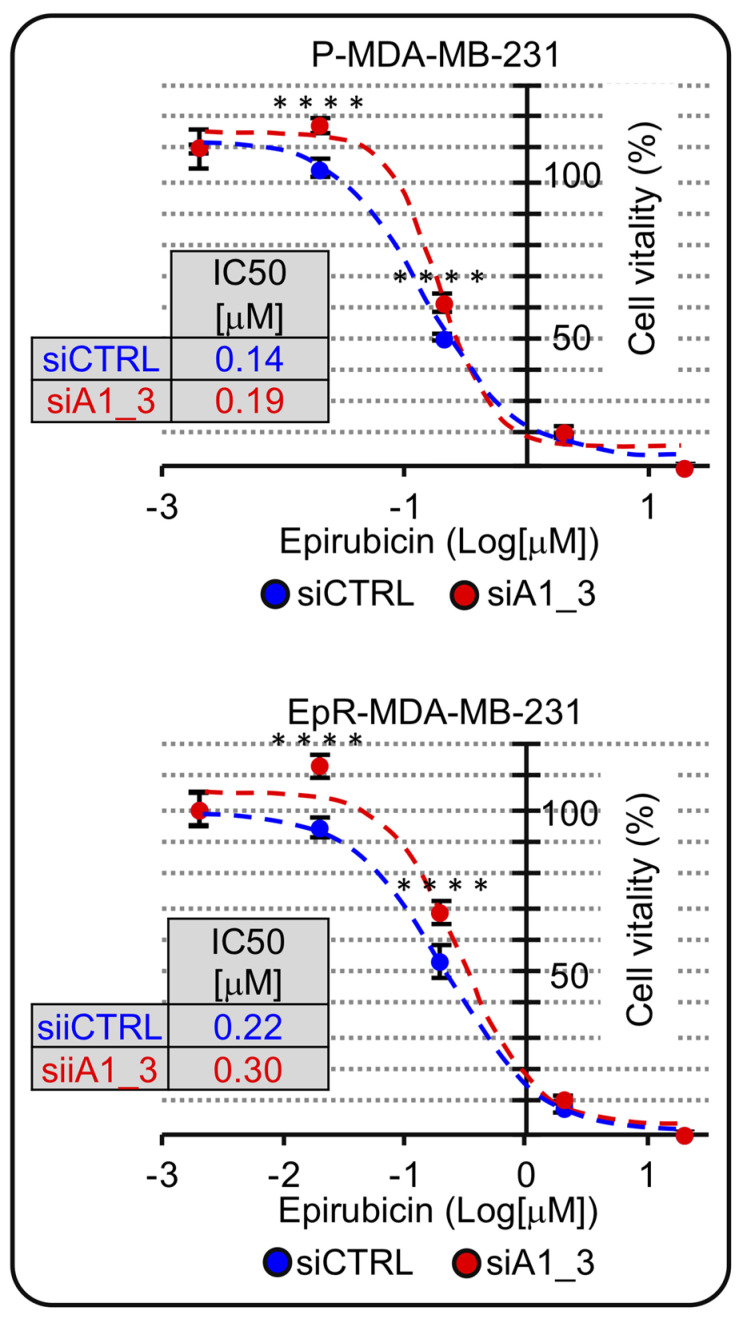
HMGA1 is a chemosensitizer factor for epirubicin-induced cytotoxicity in EpR-MDA-MB-231. MTS-assay in P-MDA-MB-231 and ER-MDA-MB-231 in HMGA1 depleted condition (siA1_3) in comparison with control condition (siCTRL) both treated with different concentration of epirubicin for 48 hrs, to define IC50 value for both cell lines in the two experimental conditions. The Sidak’s multiple comparisons test was used to define statistical significance for dose-response curve (**** = *p*_value < 0.00005).

The mechanism of action of epirubicin is linked to cell proliferation; therefore, since we noticed a change in cell-cycle distribution with an increase in the G0/G1-phase and a decrease in M-phase we hypothesized that HMGA1 silencing could negatively affect epirubicin action. We silenced HMGA1 expression in both parental and EpR cells and evaluated epirubicin IC_50_. As hypothesized, both in parental and in EpR cells, HMGA1 silencing caused an increase in the IC_50_ doses implying that HMGA1 silencing negatively affects the potency of epirubicin treatment (Figure 9). 

## 3. Discussion

A great amount of evidence supports the role of epigenetics in tumorigenesis, including BC [33]. Positional gene enrichment analyses showed that HIST1 cluster is one of the most significantly upregulated clusters of genes from the normal-like to premalignant and metastatic BC cells and tumors [34,35]. Additionally, our bioinformatic analyses highlighted in BC patients the overexpression of a subset of the RD-HIST genes, belonging to HIST1, HIST2, and HIST3 clusters. Not only histones are required for cell proliferation, but histone variants could play a relevant role as concern cancer development and acquisition of aggressiveness [36,37]. Histone gene families, belonging to HIST1 cluster, were proposed as prognostic factors for survival prediction in patients with cervical cancer [38]. Moreover, *HIST1H3F* was proposed as prognostic factor and suggested as a predictor of the prognosis in laryngeal cancer patients [39]. We found several histone genes, among those that are overexpressed in BC, having a prognostic value as concern overall survival (OS), relapse-free survival (RFS), and distant metastasis-free survival (DMFS). Four of them provide multiple prognostic data: *HIST1H4H* (RFS and DMFS), *HIST1H2BK* (OS, RFS, and DMFS), *HIST1H2BG* (RFS and DMFS) and *HIST1H1C* (OS and RFS) (Supplementary Table S2). Indeed, the overexpression of these histones is associated with a prognostic value suggesting that, besides being potential prognostic biomarkers, they could be also involved in tumor formation and development, so they could also represent potential molecular targets. Histone isoforms are very similar but not identical and the idea that they carry out different functions and roles is well accepted [40]. Obviously, all this evidence led to several questions, especially regarding their chromatin distribution and influence on chromatin related events, such as DNA accessibility, repair, and transcription. Unravelling these events could contribute to enlarging our knowledge of the strong impact of epigenetics on cancer onset and development. 

HMGA1 oncogene has been demonstrated to have a causal role in BC [13,41], its expression displays a correlation with tumor grade and promotes metastatic phenotype in TNBC [15,16]. HMGA1 is a chromatin architectural factor that constitutes a critical hub for nuclear functions. Its intrinsically disordered status confers unusual plasticity in contacting molecular partners and in exploiting transcriptional-related processes [26]. HMGA1 influences the epigenetic status of cancer cells in multiple ways. It interacts with core histones (H3, H2A, and H2B) and alters the positioning/phasing of nucleosomes onto characteristic promoter/enhancer DNA regulatory regions [42,43]. HMGA1 can positively influence both histone H3S10 phosphorylation by ribosomal protein S6 kinase alpha-3 (RSK2) and histone H2BK5 acetylation by CREB-binding protein (CBP) regulating the expression of a set of genes involved in tumor progression and EMT [44]. HMGA proteins, HMGA1 and the highly related HMGA2, compete with histone H1 for DNA-binding modulating the nucleosome accessibility resulting in a more relaxed chromatin fiber and promoting transcription [45]. We demonstrated that HMGA1 silencing leads to dephosphorylation of some histone H1 variants (*HIST1H1C*/H1.2 and *HIST1H1E*/H1.4) in TNBC cells. Moreover, we highlighted that the HMGA1-dependent increase in histone H1 phosphorylation status can affect nuclear stiffness in BC cells altering histone H1 chromatin distribution and expression suggesting that HMGA1 might affect mechanical properties of nuclei increasing plasticity and consequently invasiveness of cancer cells [46]. 

Here, we show that HMGA1 displays an additional epigenetic-related mechanism that could be relevant in BC. We provide experimental evidence that HMGA1 could play a role in the cell-cycle coordinated expression of histone genes belonging to the HIST1 cluster and that the interference of HMGA1 expression leads to significant alterations of cell-cycle distribution that are supportive of an effect in slowing down cell replication. As remarked in literature, HMGA1 dysregulation causes alterations in cell-cycle progression and cell proliferation in tumor cells [6,47,48,49,50]. In BC cells, overexpression of HMGA1 increases cell proliferation, while its downregulation has the opposite effect [13,16,51,52,53,54]. Indeed, it has been demonstrated that HMGA1 promotes the expression of several genes involved in cell-cycle regulation like *CLK1*, *CDC25A*, *CDC25B*, CCNC, and CCND1 [13,30,55,56]. Our data show that HMGA1, affecting the expression of RD-HIST, acts on a fundamental process that is intrinsically linked to the proliferative ability of cancer cells. Recently, it was shown that the other member of the HMGA family, i.e., HMGA2, is responsible for the increase in RD-HIST level in a subset of dedifferentiated liposarcoma [57]. It is therefore intriguing that in the cellular model we used several RD-HIST genes were found modulated by HMGA1.

We provide here hints of the molecular mechanisms exploited by HMGA1 to modulate histone expression focusing on the two major factors involved in histone gene expression: NPAT and SLBP. NPAT is a co-activator, recruited at the level of histone gene promoters-bound macromolecular complexes, coordinated by specific transcription factors, like octamer-binding transcription factor 1 (OCT-1) for histone H2B promoters and histone H4 transcription factor (HiNF-P) for histone H4 ones [58]. We demonstrated that HMGA1 is involved in the enhancement of *HIST1H4H* promoter activity and that HMGA1 binds directly to NPAT. We performed immunoprecipitation analyses either in the presence or absence of DNA. DNase treatment is frequently used when studying factors able to bind DNA to demonstrate that the protein/protein interaction is not mediated by DNA. Indeed, a real protein/protein interaction should be detectable both when DNA is present and upon DNase treatment. We observed the interaction only in the absence of DNA. HMGA1 is an architectural factor that can organize the formation of macromolecular complexes and can establish both DNA- and protein-interaction at the same time [28]. NPAT is one of the main transcriptional co-activators within the HLB driving histone replication-dependent histone expression [22]. HLB is a PMLO formed by a liquid-liquid phase transition process, and it is characterized by a heterogeneous and articulated network of interactions between protein and nucleic acid, either DNA or RNA [59]. Considering this information, it is therefore not surprising that NPAT/HMGA1 interaction was observed only after DNA digestion suggesting that in a very crowded molecular environment DNA could mask the antibody’s access to complexes. HMGA1 is an architectural factor that has been described as a key element in the assembly of macromolecular complexes at promoter/enhancer elements. The transcriptional-boosting activity of HMGA1 can be exerted through two main mechanisms: (i) by binding into the minor-groove of AT-rich DNA stretches causing the bending of DNA and promoting the recruitment of specific transcription factors at their own consensus sequences; (ii) by directly binding to nuclear factors influencing, thus, their conformation. Noteworthy, these two processes are not mutually exclusive and the effects of HMGA1 towards the *HIST1H4H* promoter activity could be attributed to both. We previously showed that HMGA1 regulates the expression of CCNE2 in TNBC cells [31]. Moreover, it was found that CCNE2 (and not CCNE1) associates with NPAT in the HLB of T-47D BC cells and the expression of CCNE2 correlates with RD-HISTs in BC samples [29], supporting our findings. In addition to this mechanism of regulation, we also evaluated the role of HMGA1 towards the other factor critical for histone expression regulation, SLBP. This factor binds to a stem loop located at the 3’ end of histones’ mRNA and is involved in all the aspects of histones’ RNA metabolism such as processing, nuclear export, translation, and degradation [58]. The expression of SLBP is strongly dependent upon the cell-cycle progression [25] and it is also regulated by several mechanisms involving miRNAs [60]. Thus, it would be possible that HMGA1 could regulate indirectly SLBP protein levels because of its influence on the cell-cycle progression, as highlighted by the increase in cells in the G0/G1 phase after HMGA1-silencing, and/or through the modulation of transcription of miRNA, as demonstrated for others HMGA1 targets [61,62]. Altogether, this evidence suggests a possible multi-layer mediation of HMGA1 towards the fine modulation of RD-HISTs. 

Since HMGA1 was demonstrated to affect the key event of RD-HIST expression during cell-cycle, we asked ourselves whether HMGA1 expression modulation could have an impact on the efficacy of chemotherapeutics agents acting on proliferating cells. To this aim, we developed EpR MDA-MB-231 cells since the action of this drug occurs mainly in S-phase DNA-replication [63,64]. Interestingly, we observed that the silencing of HMGA1 in EpR-MDA-MB-231 cells resulted in an increase in cells in G0/G1-phase of cell-cycle, this may suggest that HMGA1 has a role in the modulation of the cell-cycle in cells that acquired a resistance towards this drug in addition of that in parental cells. Importantly, the effect of HMGA1 silencing on cell-cycle progression has an impact on epirubicin efficacy. In fact, HMGA1, by pushing cells towards proliferation, sensitizes cells to epirubicin treatment. Indeed, our data show that the silencing of HMGA1 increases the epirubicin IC_50_ in both parental and EpR cells. This observation could be relevant for the development of combined therapeutic strategies. Moreover, this could be seen as another compelling example of an antagonistic effect between the silencing of an oncogene (i.e., HMGA1) and the use of a chemotherapeutic. This issue has been already pointed out when HMGA1 overexpression was demonstrated to act as sensitizer toward the action of cisplatin and bleomycin in BC cells [65]. Moreover, patients affected by gastric cancer and with high levels of HMGA1 expression in the tumor, had remarkably better OS when undergoing treatment with platinum- and/or fluoropirimidine-based chemotherapy [66].

In conclusion, we provide evidence that highlights a role of HMGA1 in controlling the expression of RD-HIST genes with implications in the cell-cycle of cancer cells. We are aware that our study is preliminary and that further efforts are required to better dissect at the molecular level the impact of HMGA1 on the regulation of different histone promoters and the underlying molecular mechanism(s) involving HMGA1, NPAT, and SLBP. A relevant point raised by our work is the necessity to carefully consider the potential antagonistic effect that could be detrimental for the success of a combined therapy, as showed by our results deriving by HMGA1 silencing and epirubicin treatment experiments. In other words, a modern approach may take into consideration the multilayer complexity in cell biology and cannot be based on the simple combination of two cancer-fighting strategies.

## 4. Materials and Methods

### 4.1. Cell Lines

MDA-MB-231, EpR-MDA-MB-231, HBL-100, and HEK293T cells were cultured in 10% fetal bovine serum (FBS, EuroClone, Pero (MI), Italy), 2 mM L-glutamine (EuroClone, Pero (MI), Italy), 1% penicillin/streptomycin (EuroClone, Pero (MI), Italy) in Dulbecco’s modified essential medium high glucose (DMEM HG, EuroClone, Pero (MI), Italy) at 37 °C, in a humidified 5% CO_2_ incubator (SafeGrow188, EuroClone, Pero (MI), Italy). The cells were washed with sterile PBS pH 7.4. Cells were detached using trypsin (trypsin-EDTA in PBS, EuroClone, Pero (MI), Italy). After trypsin inactivation with cell culture medium, the cell suspension was seeded according to the requested cell number.

### 4.2. Generation of EpR-MDA-MB-231 Cells

To generate EpR MDA-MB-231 cells, 3.5 x 10^6^ cells were seeded in 14 cm culture dishes and after 24 h, epirubicin was added to 0.025 µM final concentration (according to epirubicin dose-response curve). After 48 h of treatment, drug-containing media was replaced with a fresh one and was changed every 3-4 days. Within approximately 3-4 weeks, MDA-MB-231 cells recover proliferation. The procedure has been then repeated increasing progressively the epirubicin concentration (i.e., 0.025 µM, 0.050 µM, 0.075 µM, 0.1 µM, 0.125 µM). The P-MDA-MB-231 cells were grown in parallel in a dish throughout the whole procedure and exposed to vehicle with volumes corresponding to those used for epirubicin. For the functional characterization of EpR cells [resistance index (R.I.) determination] an MTS assay (Promega, Madison, WI, USA) was performed (EpR-MDA.MB-231 versus P-MDA-MB-231) using the manufacturer instruction. The semi-logarithmic curves of vitality/concentration were obtained plotting the values of absorbance (vitality) versus epirubicin concentration points (log_10_μM)

### 4.3. SiRNA Silencing

For gene silencing experiments, 5 × 10^3^ cells were seeded in a 35-mm culture dish and after 24 h the siRNA-mediated silencing was carried out using Lipofectamine (RNAiMAX, Invitrogen, Waltham, MA, USA) according to the manufacturer instruction. The siRNA mediated silencing was conducted for 72 h, after which the cells were used for protein, gene expression, MTS-assay, and flow cytometry analyses. The siRNAs used are siCTRL (5′-ACAGUCGCGUUUGCGACUGTT-3′) and siA1_3 (5′-ACUGGAGAAGGAGGAAGAGTT-3′).

### 4.4. Cell-Cycle Analysis

Cell cycle analysis was performed using propidium iodide (Sigma-Aldrich, St. Louis, MO, USA). After 72 h of silencing, EpR-, P- and MDA-MB-231 cells were collected by centrifugation, washed with cold PBS, and fixed with cold 70% Ethanol overnight (O.N.). The day after, the cell pellet was centrifuged, washed two times with cold PBS, and incubated with PI (10 μg/mL) in PBS O.N. Cells were analyzed with a flow cytometer. Measurements were carried out with a Attune NxT Flow Cytometer (Thermo Scientific, Waltham, MA, USA) characterized by acoustic focusing technology, equipped with flat-top Blue laser (488 nm, 50 mW) with wavelength-tuned photomultiplier tubes and standard configuration (4 channel colors). After acquisition of at least 10,000 events for each run, maintaining flow rates at 25 μL/min, data were stored as FCS files and analyzed with the FCS express V7 (Muticycle-DNA) De Novo Software.

### 4.5. Plasmid Transfection and Luciferase Assay

Plasmids used for transfections are listed below: pEGFP-N1, pEGFP-N1 HMGA1a, pRL-CMV Renilla (Promega, Madison, WI, USA) and pGL4.11 (Promega, Madison, WI, USA) were already present in the laboratory; pGL4.11-H4Hprom (−950, +50) containing a region of the HIST1H4H promoter spanning from −950 to +50 bp was generated by amplifying HIST1H4H promoter from a female healthy total DNA with the forward primer 5′- GGC CGC GGT ACC GTA ATT TAA GAA AA -3′ and the reverse primer 5′- CCA AGC TTC CTT ACC CAA ACC TTT TCC -3′. Subsequently, the PCR product was cloned in the pGL4.11 vector (GE HealthCare, Chicago, IL, USA) using *Kpn*I and *Hind*III restriction enzymes. Plasmid transfections were carried out using the standard calcium phosphate transfection method. Briefly, 3.5 × 10^5^ HEK293T cells were seeded in 35-mm-diameter culture dishes the day before the transfection and processed 40 h after transfection. A total amount of 1.02 μg plasmid DNA was transfected in HEK293T cells. Specifically, the following amounts of plasmids were used: 100 ng of the Luciferase reporter (pGL4.11-H4H); 800 ng of pEGFP-N1 in the control condition for the pGL4.11-H4H; 100 ng of pEGFP-N1 HMGA1a. 20 ng of pRL-CMV Renilla luciferase was used as normalizer for transfection efficiency. The Dual-Luciferase^®^ Reporter Assay System (Promega, Madison, WI, USA) was used for the luciferase reporter assay, following the manufacturer instructions. The measurements were carried out using the Berthold Lumat LB 9501 Tube Luminometer.

### 4.6. Co-Immunoprecipitation

MDA-MB-231 cells protein extract was prepared in an IP buffer as already described [44]. For the Co-IP experiment, G proteins agarose beads (GE HealthCare, Chicago, IL, USA) were incubated with 4 μg of either α-HMGA1 [44] or α-GFP (GTX113617 Genetex, Irvine, CA, USA), as a negative control. Agarose beads were blocked with 1 mg/mL bovine serum albumin (BSA) (Sigma-Aldrich, St. Louis, MO, USA) for 1 h at 4 °C under rotation. 550 μg of cell lysate was digested with 1100 U DNase I for 20 min at 37 °C. 50 μg of this lysate was incubated with RNAase A (10 μg) for 1 h at 55 °C in 1% sarcosyl and 25 mM EDTA buffer and treated with Proteinase K (23 μg) for 1h at 55 °C. This aliquot was loaded on 1% agarose gel to verify DNA digestion. 500 μg of cell lysate (both DNaseI treated and not treated) were incubated with G protein agarose beads-antibody complex (α-HMGA1 or α-GFP) in Lysis Buffer for 3 h at 4 °C under rotation. Beads were washed three times in Tris/HCl pH seven, and proteins were eluted by boiling the beads for 5 min in SDS-buffer and analyzed by western blot with the indicated antibodies.

### 4.7. SDS Polyacrylamide Gel Electrophoresis (SDS-PAGE) and Western Blot Analyses

Nuclear extracts were obtained by lysing cells in N-buffer (Tris/HCl pH 7.5 15 mM, KCl 60 mM, NaCl 15 mM, MgCl_2_ 5 mM, CaCl_2_ 1 mM NaVanadate 2 mM, sucrose 250 mM, 0.3% NP-40) and passed 5 times through a 21G needle. Nuclei were recovered by centrifugation (2000× *g* 5 min) and analyzed in SDS lysis buffer. For total cell extracts, cells were washed in ice-cold PBS and lysed within the plate with SDS sample buffer supplemented with proteases inhibitors. The cells were scraped, the lysed material was recovered and separated by SDS-PAGE. For gel staining, after SDS-PAGE, gels were soaked in a solution containing water/methanol/acetic acid (4:5:1) and 0.05% *w*/*v* Coomassie Blue O.N. and de-stained in a 10% *v*/*v* acetic acid solution. Gel images were acquired using ChemiDoc™ Touch Imaging System or ImageScanner UMAX with Image Lab™ Touch Software or ImageMaster 2D v4.01 software to determine the total intensity of each lane that was used for quantification/normalization analyses. Western blot analyses were carried out under standard procedures using nitrocellulose membranes (GE HealthCare, Chicago, IL, USA) and the following primary antibodies: α-HMGA1 [44], α-GFP (GTX113617, Genetex, Irvine, CA, USA), α-SLBP (ab181972, Abcam, Cambridge, UK), α-NPAT (LS-C289483, LSBio, Seattle, WA, USA), CyclinE2 (ab226388, Abcam, Cambridge, UK), and α-Cdk2 (ab235941, Abcam, Cambridge, UK). For detection, a secondary antibody conjugated with Horseradish Peroxidase (HRP) was used, membranes were then treated with an Enhanced Chemiluminescence solution (ECL Western Blotting Substrate, Pierce, Rockford, IL, USA) for chemiluminescence detection (with autoradiography films (GE HealthCare, Chicago, IL, USA) or with ChemiDoc™ Touch Imaging System).

### 4.8. RNA Extraction and RT-qPCR

Total RNA extraction was achieved using Trizol (EuroClone, Pero (MI), Italy) following the manufacturer instructions. The isolated RNA was subjected to DNase I (Invitrogen, Waltham, MA, USA) treatment and to a subsequent phenol/chlorophorm purification. The quality of the RNA extracted was assessed by agarose gel electrophoresis in denaturing conditions. RNA concentration was measured with a Nanodrop 2000 spectrophotometer (Thermo Scientific, Waltham, MA, USA). cDNA was obtained by retrotranscription using random primers and by Superscript III (Invitrogen, Waltham, MA, USA) according to the manufacturer’s instructions. The CFX96 Real Time PCR system (Biorad, Hercules, CA, USA) was used to perform quantitative Polymerase Chain Reaction (qPCR). The reaction mix was made with IQ SYBR Green Supermix 2X (Biorad, Hercules, CA, USA). The thermal cycling protocol was: 5 min at 95 °C, 40 cycles of amplification composed by 5 s at 95 °C followed by 45 s at 60 °C, finally a melting curve analysis was obtained by ramping the temperature from 60 to 95 °C (1 °C/10 s). Data were analyzed by Biorad CFX Manager software (Biorad, Hercules, CA, USA) and relative gene expression was calculated by the ΔΔCt method, using the GAPDH as normalizer. The sequence of primers used for RT-qPCR is reported in Table 1.

### 4.9. Bioinformatics and Statistical Analysis

The differential gene expression and correlation analysis was performed comparing common genes between the SCAN-B dataset (GSE202203) and normal breast tissue obtained from the GTEx Portal (dbGaP Accession phs000424.v8.p2). In order to eliminate the batch effect deriving from the merging of the datasets, the RUVSeq R/Bioconductor package [67] was used in combination with the R/Bioconductor DESeq2 package [68]. The merged expression model was used to perform the differential expression (DE) analysis using DESeq2. Differentially expressed genes (DEGs) were selected with a Log2Fold Change value greater than 1 or lower than −1 and with a P-adjusted value cut off of 0.05. The P-value was adjusted for multiple testing using the Benjamini–Hochberg (BH) correction with a false discovery rate (FDR) ≤ 0.05. The analyses were performed either comparing all the tumoral SCAN-B samples versus normal ones or considering the following BC subtype included in the SCAN-B dataset: basal, luminal A, luminal B, HER2. Samples labelled as normal-like and unclassified were excluded from correlation analysis. RD-HIST gene list [21] is composed of 72 genes but only 63 of them were mapped to the transcription profiles and thus included in the analyses. Out of those 63 genes, 58 RD-HIST genes were differentially expressed in at least one subtype (RD-HIST-R gene list).

The correlation analysis for HMGA1 was performed using the normalized count matrix (obtained from variance stabilizing transformation (VST) method as implemented in DESeq2 package) and P-value was adjusted for multiple testing with BH correction with a FDR  ≤ 0.05. Genes were selected with a P-adjusted value cut off of 0.05. In order to plot the distributions of histones’ correlations R/ggpubr package was used. As a control, randomly selected genes were chosen excluding the RD-HIST-R genes. In order to test the statistical support of the different correlations, two Empirical Cumulative Distribution Functions (ECDFs) were generated for each comparison and tested with the Kolmogorov-Smirnov test.

Prognostic data were obtained with KM plotter (http://kmplot.com/analysis (accessed on 4 July 2022)). The “mRNA breast cancer” gene chip database was used for KM plotter. The patients were split by median and OS (Overall Survival), DMFS (Distant Metastasis Free Survival), and RFS (Relapse Free Survival) were assayed over a period of 300 months (25 years). KM plotter has automatically provided the Hazard Ratio (HR), the 95% confidence interval, and determined the statistical significance for each analysis.

Statistical analyses performed to assess differences in expression levels between samples were done using a two tailed Student’s *t*-test. The Sidak’s multiple comparisons test was used for the dose response curves.

## Figures and Tables

**Table 1:** List of primers employed for RT-qPCR.

Oligo Name	Sequence 5′ → 3′	Oligo Name	Sequence 5′ → 3′
GAPDH Fw	TCTCTGCTCCTCCTGTTC	GAPDH Rev	GCCCAATACGACCAAATCC
HMGA1 Fw	ACCAGCGCAAATGTTCATCCTCA	HMGA1 Rev	AGCCCCTCTTCCCCACAAAGAGT
SLBP Fw	GGAAGAACACAATTGCCTACG	SLBP Rev	TAGGGGTCTTGGGATGAATG
HIST1H1C Fw	CACCGAAGAAAGCGAAGAAG	HIST1H1C Rev	AGCCTTAGCAGCACTTTTGG
HIST1H2AC Fw	ACGAGGAGCTCAACAAACTG	HIST1H2AC Rev	GTCAAATCACTTGCCCTTGG
HIST1H2BD Fw	TAACGCTACGATGCCTGAAC	HIST1H2BD Rev	TTCTTCCCGTCCTTCTTCTG
HIST1H4H Fw	CCGTGGTAAAGGTGGAAAAG	HIST1H4H Rev	GCCAGAAATTCGCTTGACAC

Fw: Forward; Rev: Reverse.

## Supplementary Materials

The following supporting information can be downloaded at: https://www.mdpi.com/article/10.3390/ijms24010594/s1.
