# Supplementary material for: HMGA1 Regulates the Expression of Replication-Dependent Histone Genes and Cell-Cycle in Breast Cancer Cells
Source: Int J Mol Sci. 2022 Dec 29;24(1):594. doi: 10.3390/ijms24010594 (PMC9820469; doi:10.3390/ijms24010594)
Supplement: Supplementary file 1 [file ijms-24-00594-s001.zip › Supplementary figures final 21_11.pdf]

Supplementary figures

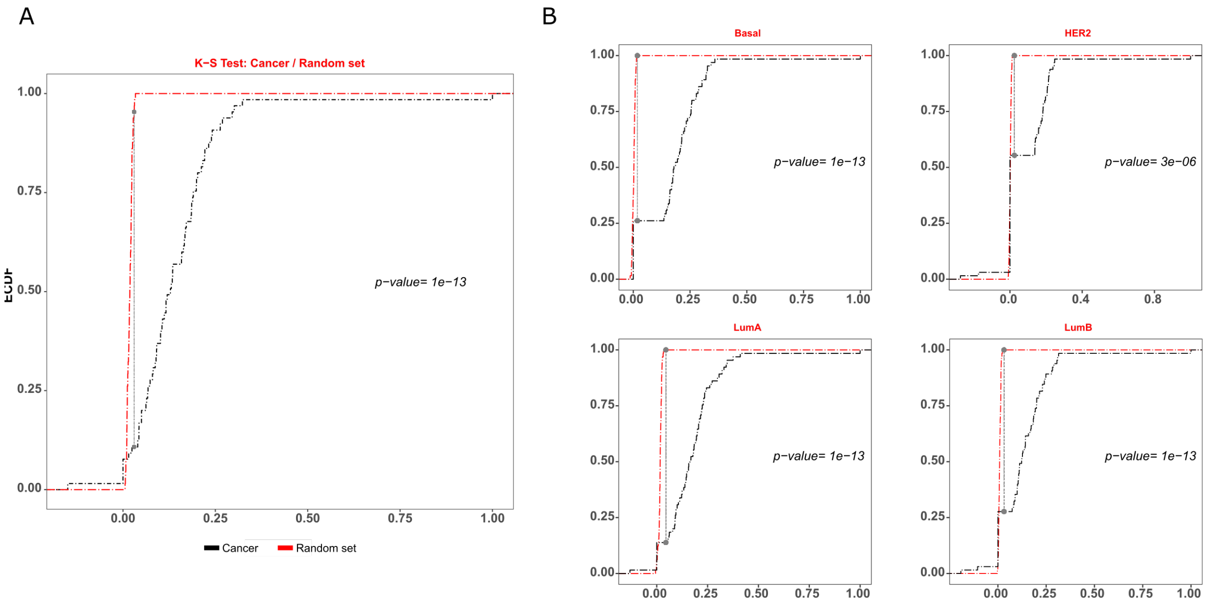

Figure S1. A. Empirical Cumulative Distribution Functions (ECDFs) of all Breast Cancer subtypes (black dashed line) and its random set (red dashed line). The correlations between HMGA1 and RD-HIST-R genes were compared with the Kolmagorov-Smirnov test and found to be statistically significant (p-value = 1.00×10^{-13}). Highlighted points in gray and their connecting dotted line show the highest and lowest points between the two ECDFs.

B. Empirical Cumulative Distribution Functions (ECDFs) of all Breast Cancer subtypes (black dashed line) and its random set (red dashed line). The correlations between HMGA1 and RD-HIST-R genes were compared, and all found to be statistically significant. Highlighted points in grey and their connecting dotted line show the highest and lowest points between the two ECDFs.

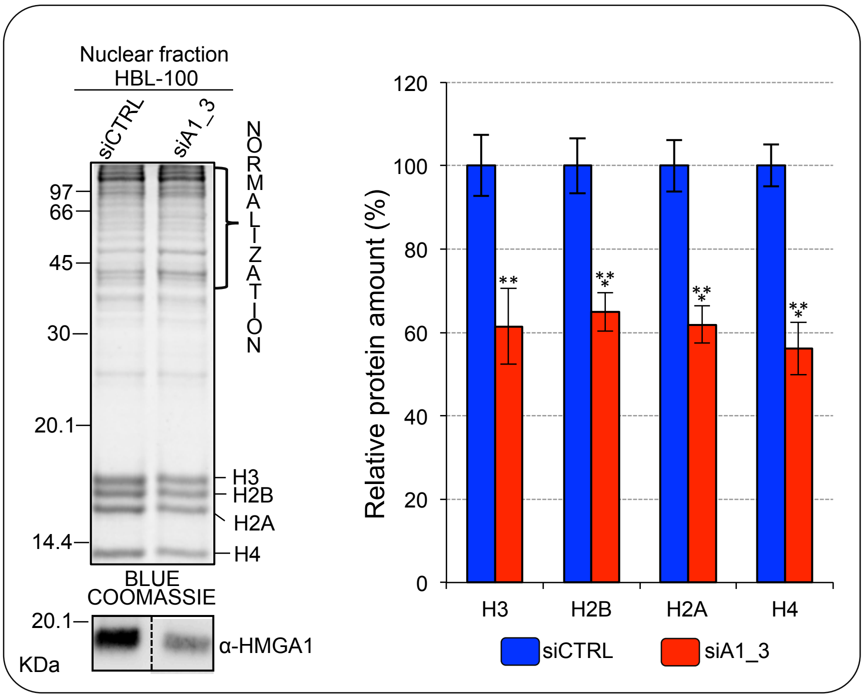

Figure S2. HMGA1 regulates core histones protein expression in HBL-100 cells. Blue Coomassie stained gel was used to assess core histones protein amount in the nuclear fraction of HBL-100 cells silenced for HMGA1 (siA1_3) and with control siRNA (siCTRL). The representative Blue Coomassie stained gel, on the left, is shown together with molecular weight markers (kDa). The upper part of the gel (not including histones bands) was exploited for total protein normalization. A western blot - on the left, below –performed with α-HMGA1 antibody is shown to assess the silencing of HMGA1 expression. The histogram graph, on the right, was obtained with densitometric analyses (siCTRL versus siA1_3) of protein staining. The upper part of the stained gel was used to normalize core histones total amount. Bars indicate the means. Standard deviations are shown (n=4). Statistical significance was assessed with Student's t test (*: p≤ 0.05; **: p≤0.01; ***: p≤0.001).

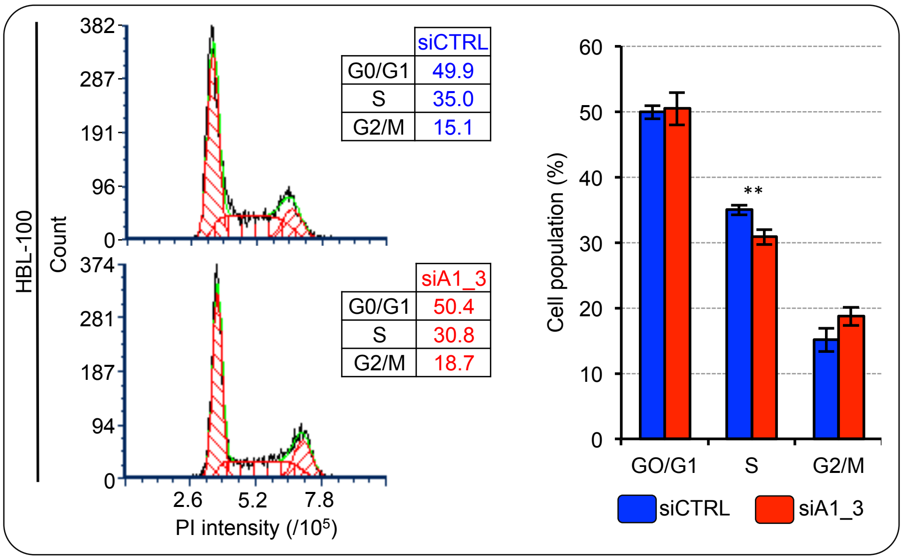

Figure S3. HMGA1 depletion influences HBL-100 cell-cycle progression. Representative graphs showing the staining with PI of cells silenced for HMGA1 (siA1_3), lower left part, or treated with control siRNA (siCTRL), upper left part. The histogram graph on the right shows the relative percentage count of MDA-MB-231 cells in G0/G1-, S- and G2/M-phase of cell cycle for both conditions (siCTRL and siA1_3). Bars indicate the means. Standard deviations are shown (n=3). Statistical significance was assessed with Student's t test (*: p≤0.05; **: p≤0.01; ***: p≤0.001).

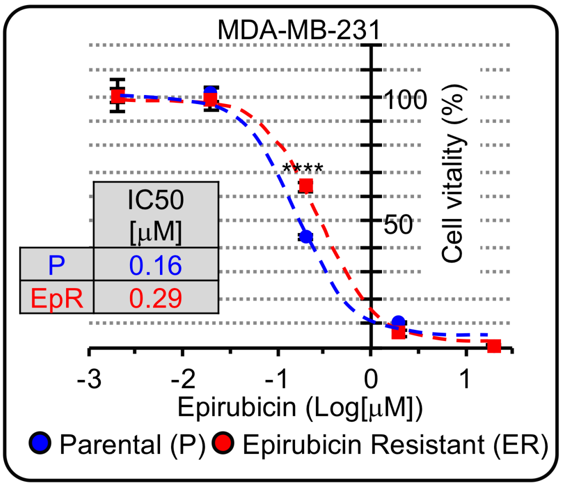

Figure S4. Functional characterization of EpR-MDA MB-231 cells. To define resistance index (R.I. = $IC50_{EpR\text{-}cells}/IC50_{P\text{-}ells}$ =2.1), EpR-MDA MB-231 cells were treated with increasing epirubicin concentrations [$\log_{10}$ (0.002 μM)=-2.7, $\log_{10}$ (0.02

μM)=-1.7, $\log_{10}$ (0.2 μM)=-0.7, $\log_{10}$ (2 μM)=0.3 and $\log_{10}$ (20 μM)=1.3)] for 48h. Control cells were treated with an equivalent methanol volume. For dose-response curve determination a metabolic activity assay (MTS assay) has been used to evaluate cell vitality of EpR-MDA-MB-231 compared to parental cells (P-). Dots and squares indicate the mean values. Standard deviations are shown (n=4). The Sidak's multiple comparisons test was used to define statistical significance for dose-response curve (*=p_value<0.05, **=p_value<0.005, ***=p_value<0.0005, ****=p_value<0.00005).
