# Supplementary material for: HMGA1 Regulates the Expression of Replication-Dependent Histone Genes and Cell-Cycle in Breast Cancer Cells
Source: Int J Mol Sci. 2022 Dec 29;24(1):594. doi: 10.3390/ijms24010594 (PMC9820469; doi:10.3390/ijms24010594)
Supplement: Supplementary file 1 [file ijms-24-00594-s001.zip › Supplementary Table S1.pdf]

Table S1. Differential expressed RD-HIST genes. Log2FC and P-adj (P-value adjusted for multiple testing using the Benjamini–Hochberg (BH)) for each RD-histone gene and HMGA1 for all subtypes or each BC subtype compared to healthy tissue. Genes that were not differentially expressed (P > 0.05 & |log2FC| < 1) are labelled as NDE.

Differential Expression of RD-histones in BC subtypes											
		Basal (n = 317)		HER2E (n = 278)		LumA (n = 1540)		LumB (n = 896)		All subtypes (n = 3207)	
Genes	Ensembl ID	log_2FC	*P-adj*	log_2FC	*P-adj*	log_2FC	*P-adj*	log_2FC	*P-adj*	log_2FC	*P-adj*
HMGA1	ENSG00000137309	3.11	0	2.39	0	1.14	05.90 x 10^{-265}	1.71	02.57 x 10^{-274}	1.86	0
HIST1H1A	ENSG00000124610	5.32	06.09 x 10^{-179}	3.7	01.81 x 10^{-47}	1.53	03.54 x 10^{-20}	1.8	01.19 x 10^{-11}	2.93	08.75 x 10^{-58}
HIST1H1B	ENSG00000184357	3.98	05.32 x 10^{-191}	3.6	06.16 x 10^{-152}	2.05	09.76 x 10^{-109}	3.57	04.20 x 10^{-193}	3.02	04.38 x 10^{-262}
HIST1H1C	ENSG00000187837	1.27	05.62 x 10^{-48}	1.97	03.42 x 10^{-105}	1	01.07 x 10^{-48}	1.79	06.15 x 10^{-97}	1.54	07.09 x 10^{-104}
HIST1H1D	ENSG00000124575	1.32	07.21 x 10^{-33}	1.78	03.55 x 10^{-50}	NDE		2.28	02.85 x 10^{-96}	1.08	03.70 x 10^{-45}
HIST1H1E	ENSG00000168298	NDE		NDE		NDE		NDE		NDE	
HIST1H1T	ENSG00000187475	2.83	02.79 x 10^{-26}	2.78	08.75 x 10^{-21}	2.55	02.31 x 10^{-35}	3.2	02.43 x 10^{-45}	2.91	04.02 x 10^{-57}
HIST1H2AA	ENSG00000164508	NDE		NDE		NDE		NDE		NDE	
HIST1H2AB	ENSG00000278463	2.51	07.17 x 10^{-38}	1.8	04.50 x 10^{-17}	NDE		1.4	04.38 x 10^{-16}	1.08	01.54 x 10^{-16}
HIST1H2AC	ENSG00000180573	1.17	03.07 x 10^{-50}	1.21	05.94 x 10^{-48}	1.6	01.89 x 10^{-191}	1.82	03.40 x 10^{-135}	1.46	05.31 x 10^{-167}
HIST1H2AD	ENSG00000196866	NDE		NDE		NDE		NDE		NDE	
HIST1H2AE	ENSG00000277075	3.26	09.49 x 10^{-208}	4.07	02.35 x 10^{-218}	1.99	02.75 x 10^{-152}	3.63	02.64 x 10^{-242}	2.32	04.24 x 10^{-198}
HIST1H2AG	ENSG00000196787	3.36	08.11 x 10^{-304}	3.78	01.20 x 10^{-257}	2.52	09.54 x 10^{-196}	3.53	01.90 x 10^{-234}	3.09	08.48 x 10^{-298}
HIST1H2AH	ENSG00000274997	1.8	08.14 x 10^{-42}	NDE		NDE		1.02	01.24 x 10^{-16}	NDE	
HIST1H2AI	ENSG00000196747	5.89	0	6.87	0	5.57	0	7.33	0	6.19	0
HIST1H2AJ	ENSG00000276368	6.09	0	6.63	0	5.21	0	6.65	0	6.14	0
HIST1H2AL	ENSG00000276903	4.09	01.94 x 10^{-143}	3.99	01.64 x 10^{-137}	2.08	01.36 x 10^{-77}	3.47	01.16 x 10^{-155}	3.01	05.69 x 10^{-189}
HIST1H2AM	ENSG00000278677	8.81	0	9.14	0	7.69	0	8.52	0	8.46	0
HIST1H2BA	ENSG00000146047	NDE		NDE		NDE		NDE		NDE	
HIST1H2BB	ENSG00000276410	3.47	01.11 x 10^{-69}	3.03	06.78 x 10^{-50}	1.82	05.25 x 10^{-38}	2.94	02.92 x 10^{-63}	2.59	02.54 x 10^{-85}
HIST1H2BC	ENSG00000180596	2.27	06.57 x 10^{-142}	3.03	01.41 x 10^{-204}	2.73	01.25 x 10^{-287}	3.34	06.83 x 10^{-299}	2.81	0
HIST1H2BD	ENSG00000158373	2.18	05.84 x 10^{-138}	2.59	01.76 x 10^{-171}	2.43	0	3.62	0	2.49	0
HIST1H2BE	ENSG00000274290	3.72	0	4.11	0	3.74	0	4.67	0	3.95	0
HIST1H2BF	ENSG00000277224	2.26	04.97 x 10^{-56}	2.57	03.15 x 10^{-62}	NDE		3.4	01.02 x 10^{-164}	1.18	07.85 x 10^{-45}
HIST1H2BG	ENSG00000273802	1.59	09.78 x 10^{-40}	2.54	02.92 x 10^{-79}	NDE		2.84	09.03 x 10^{-121}	NDE	
HIST1H2BH	ENSG00000275713	2.81	01.02 x 10^{-106}	2.93	05.48 x 10^{-97}	NDE		2.99	01.55 x 10^{-126}	1	01.15 x 10^{-32}
HIST1H2BI	ENSG00000278588	2.03	03.97 x 10^{-37}	2.02	01.38 x 10^{-35}	NDE		1.86	01.27 x 10^{-36}	1.38	05.03 x 10^{-42}
HIST1H2BJ	ENSG00000124635	3.4	03.95 x 10^{-286}	3	01.02 x 10^{-211}	1.6	05.68 x 10^{-160}	2.55	01.27 x 10^{-203}	2.33	08.67 x 10^{-271}
HIST1H2BK	ENSG00000197903	3.15	0	3.21	0	2.1	0	2.78	0	2.69	0
HIST1H2BL	ENSG00000185130	3.57	01.31 x 10^{-155}	3.83	01.46 x 10^{-169}	2.12	01.16 x 10^{-90}	3.44	01.25 x 10^{-165}	2.93	08.06 x 10^{-195}
HIST1H2BM	ENSG00000273703	2.78	09.02 x 10^{-38}	2.87	01.47 x 10^{-40}	NDE		2.62	03.29 x 10^{-38}	1.83	09.74 x 10^{-36}
HIST1H2BN	ENSG00000233822	1.68	02.08 x 10^{-144}	1.42	04.53 x 10^{-111}	NDE		1.79	08.99 x 10^{-156}	1.05	08.36 x 10^{-89}
HIST1H2BO	ENSG00000274641	5.19	03.55 x 10^{-274}	4.96	01.17 x 10^{-239}	3.24	04.60 x 10^{-216}	4.57	04.42 x 10^{-303}	4.15	0
HIST1H3A	ENSG00000275714	1.75	01.37 x 10^{-39}	2.08	02.79 x 10^{-54}	NDE		NDE		1.07	03.57 x 10^{-27}
HIST1H3B	ENSG00000286522	5.91	07.19 x 10^{-301}	5.81	09.68 x 10^{-271}	3.7	01.37 x 10^{-176}	5.13	01.04 x 10^{-245}	4.93	0
HIST1H3C	ENSG00000287080	4.24	01.39 x 10^{-146}	4.41	02.16 x 10^{-162}	2.49	01.28 x 10^{-92}	3.84	02.40 x 10^{-145}	3.57	06.18 x 10^{-194}
HIST1H3D	ENSG00000197409	4.05	03.34 x 10^{-227}	4.38	06.78 x 10^{-232}	3.05	01.09 x 10^{-211}	5.03	0	3.32	04.94 x 10^{-288}
HIST1H3E	ENSG00000274750	1.74	02.59 x 10^{-74}	1.87	02.90 x 10^{-81}	1.48	04.00 x 10^{-128}	2.1	01.85 x 10^{-119}	1.5	01.80 x 10^{-130}
HIST1H3F	ENSG00000277775	4.24	04.33 x 10^{-133}	4.18	06.32 x 10^{-102}	2.34	01.21 x 10^{-56}	3.74	05.42 x 10^{-104}	3.31	05.54 x 10^{-124}
HIST1H3G	ENSG00000273983	4.29	03.93 x 10^{-168}	5.51	04.51 x 10^{-222}	3.02	09.37 x 10^{-189}	4.31	04.28 x 10^{-179}	3.9	03.23 x 10^{-259}
HIST1H3H	ENSG00000278828	3.36	01.21 x 10^{-223}	4.59	0	3.26	07.79 x 10^{-271}	4.73	0	3.82	0
HIST1H3I	ENSG00000275379	3.17	08.77 x 10^{-65}	2.77	01.34 x 10^{-41}	1.52	04.39 x 10^{-27}	2.27	05.80 x 10^{-32}	2.16	03.11 x 10^{-60}
HIST1H3J	ENSG00000197153	5.25	06.01 x 10^{-234}	5.29	09.73 x 10^{-208}	3.26	01.45 x 10^{-138}	4.94	08.30 x 10^{-234}	4.33	02.16 x 10^{-263}
HIST1H4A	ENSG00000278637	3.15	05.36 x 10^{-91}	3.12	08.28 x 10^{-85}	2.24	04.36 x 10^{-93}	3.08	01.59 x 10^{-95}	2.75	01.20 x 10^{-155}
HIST1H4B	ENSG00000278705	1.31	09.89 x 10^{-17}	1.31	02.01 x 10^{-15}	NDE		1.04	01.02 x 10^{-14}	1.04	03.19 x 10^{-31}
HIST1H4C	ENSG00000197061	7.34	0	6.74	0	5.73	0	6.58	0	6.46	0
HIST1H4D	ENSG00000277157	2.66	02.96 x 10^{-65}	2.83	06.75 x 10^{-70}	2.1	04.73 x 10^{-77}	3	07.98 x 10^{-107}	2.53	02.34 x 10^{-127}
HIST1H4E	ENSG00000276966	1.29	03.65 x 10^{-33}	1.22	02.19 x 10^{-26}	NDE		1.57	01.63 x 10^{-55}	NDE	
HIST1H4F	ENSG00000274618	3.79	06.04 x 10^{-70}	3.34	06.85 x 10^{-46}	2.62	04.04 x 10^{-65}	3.14	01.29 x 10^{-48}	3.27	01.85 x 10^{-105}
HIST1H4G	ENSG00000275663	NDE		NDE		NDE		NDE		NDE	
HIST1H4H	ENSG00000158406	1.68	07.22 x 10^{-84}	2.96	08.94 x 10^{-205}	1.32	03.15 x 10^{-69}	2.25	03.35 x 10^{-137}	1.84	01.11 x 10^{-128}
HIST1H4I	ENSG00000276180	NDE		NDE		-1.65	02.16 x 10^{-150}	-1.02	02.09 x 10^{-37}	-1.16	01.02 x 10^{-68}
HIST1H4J	ENSG00000197238	6.14	0	6.79	0	6.15	0	6.69	0	6.57	0
HIST1H4K	ENSG00000273542	6.56	0	7.09	0	6.28	0	7.1	0	6.63	0
HIST1H4L	ENSG00000275126	2.9	07.85 x 10^{-25}	1.77	04.93 x 10^{-5}	1.37	07.75 x 10^{-10}	1.33	04.47 x 10^{-5}	1.7	02.81 x 10^{-18}
HIST2H2AB	ENSG00000184270	1.86	08.59 x 10^{-66}	2.14	01.02 x 10^{-83}	1.01	07.48 x 10^{-44}	2.3	07.31 x 10^{-124}	1.72	01.73 x 10^{-132}
HIST2H2AC	ENSG00000184260	2.7	09.71 x 10^{-240}	3.13	02.71 x 10^{-300}	2.17	0	3.29	0	2.76	0
HIST2H2BE	ENSG00000184678	NDE		1.09	01.36 x 10^{-36}	NDE		2.01	02.06 x 10^{-133}	NDE	
HIST2H3A	ENSG00000203852	7.08	0	7.49	0	5.08	0	6.72	0	6.32	0
HIST2H3C	ENSG00000203811	7.32	0	7.71	0	5.3	0	6.88	0	6.56	0
HIST3H2A	ENSG00000181218	2.9	0	2.79	07.06 x 10^{-273}	1.83	02.49 x 10^{-170}	3.07	02.01 x 10^{-198}	2.31	01.68 x 10^{-232}
HIST3H2BB	ENSG00000196890	2	01.00 x 10^{-84}	2.88	06.23 x 10^{-148}	1.88	01.89 x 10^{-95}	3.46	09.84 x 10^{-168}	2.2	01.17 x 10^{-132}
HIST3H3	ENSG00000168148	-1.93	02.25 x 10^{-26}	1.1	01.84 x 10^{-5}	-2.34	04.00 x 10^{-27}	-1.88	04.10 x 10^{-21}	-1.82	02.41 x 10^{-7}
HIST4H4	ENSG00000197837	-1.53	02.41 x 10^{-134}	-1.15	02.34 x 10^{-75}	-1.11	02.97 x 10^{-104}	NDE		NDE	
