# Supplementary material for: HMGA1 Regulates the Expression of Replication-Dependent Histone Genes and Cell-Cycle in Breast Cancer Cells
Source: Int J Mol Sci. 2022 Dec 29;24(1):594. doi: 10.3390/ijms24010594 (PMC9820469; doi:10.3390/ijms24010594)
Supplement: Supplementary file 1 [file ijms-24-00594-s001.zip › Supplementary Table S2.pdf]

Table S2. Prognostic data from Kaplan-Meier PLOTTER of Histone genes expression in BC.

(B) Gene Symbol	Affy ID	OVERALL SURVIVAL		RELAPSE-FREE SURVIVAL		DISTANT METASTASIS-FREE SURVIVAL		Affy ID targets (with synonyms)
		HR	P	HR	P	HR	P	
HIST1H1A	208484_at	1.12 (0.93-1.35)	0.23	**0.87 (0.79-0.96)**	**0.007**	1.09 (0.93-1.27)	0.28	HIST1H1A, H1.1, H1F1
HIST1H1B	214534_at	0.96 (0.79-1.15)	0.64	0.93 (0.84-1.02)	0.13	1.03 (0.89-1.21)	0.68	HIST1H1B, H1.5, H1F5
HIST1H1C	209398_at	**1.31 (1.09-1.58)**	**0.004**	**1.18 (1.07-1.3)**	**0.0014**	1.02 (0.88-1.19)	0.77	HIST1H1C, H1F2, H1.2
HIST1H1D	214537_at	1.1 (0.91-1.32)	0.34	1.02 (0.92-1.13)	0.68	1.04 (0.89-1.21)	0.62	HIST1H1D, H1F3, H1.3
HIST1H1E	208552_at	1.16 (0.96-1.4)	0.12	**1.16 (1.05-1.28)**	**0.0044**	1.07 (0.91-1.24)	0.42	HIST1H1E, H1F4, H1.4
HIST1H1T	1553568_a_a	0.87 (0.66-1.13)	0.29	**0.7 (0.6-0.82)**	**4.3e-06**	0.84 (0.65-1.1)	0.21	HIST1H1T
	207982_at	0.99 (0.82-1.19)	0.89	1.02 (0.92-1.12)	0.76	**1.18 (1.01-1.37)**	**0.038**	H1t, H1FT, HIST1H1T
HIST1H2AA	-	-	-	-	-	-	-	-
HIST1H2AB	208569_at	**1.29 (1.07-1.56)**	**0.007**	**0.88 (0.8-0.97)**	**0.013**	1.13 (0.97-1.32)	0.13	HIS1H2AB, H2A/m, H2AFM
HIST1H2AC	215071_s_at	1.12 (0.93-1.36)	0.22	0.98 (0.89-1.08)	0.69	0.93 (0.8-1.08)	0.35	HIST1H2AC, H2AFL
HIST1H2AE	214469_at	1.2 (0.99-1.44)	0.061	1.08 (0.98-1.19)	0.14	1.01 (0.87-1.18)	0.89	HIST1H2AE, H2A.1, H2A/a, H2AFA
HIST1H2AG	207156_at	1.08 (0.89-1.3)	0.45	**1.14 (1.03-1.26)**	**0.013**	1.14 (0.98-1.33)	0.095	HIST1H2AG, H2A/p, H2A.1b, pH2Af, H2AFP
HIST1H2AH	-	-	-	-	-	-	-	-
HIST1H2AI	214542_x_at	0.94 (0.78-1.14)	0.52	0.93 (0.84-1.03)	0.17	1.02 (0.87-1.19)	0.83	HIST1H2AI, H2AFC, H2A/c
HIST1H2AJ	208583_x_at	0.94 (0.78-1.13)	0.52	0.93 (0.84-1.02)	0.13	1.01 (0.86-1.17)	0.93	HIST1H2AJ, H2A/E, H2AFE
HIST1H2AK	214644_at	0.94 (0.78-1.13)	0.49	0.95 (0.86-1.05)	0.31	0.95 (0.82-1.11)	0.56	HIST1H2AK, H2AFD, H2A/d
HIST1H2AL	214554_at	1.07 (0.88-1.29)	0.5	0.98 (0.89-1.09)	0.75	1.09 (0.93-1.27)	0.27	HIST1H2AL, dj193B12.9, H2A/i, H2AFI
HIST1H2AM	214481_at	1.16 (0.96-1.4)	0.13	**1.12 (1.02-1.24)**	**0.023**	1.1 (0.94-1.28)	0.25	HIST1H2AM, H2A.1, H2A/n, H2AFN
HIST1H2BA	241519_at	1.09 (0.83-1.43)	0.53	0.87 (0.75-1.01)	0.064	1.09 (0.84-1.42)	0.52	HIST1H2BA
HIST1H2BB	208547_at	1.06 (0.88-1.28)	0.55	**1.23 (1.11-1.36)**	**4.5e-05**	1.02 (0.87-1.19)	0.82	HIST1H2BB, H2BFF, H2B/f
HIST1H2BC	214455_at	1.14 (0.95-1.38)	0.16	1.04 (0.94-1.15)	0.48	1.02 (0.88-1.19)	0.77	HIST1H2BC, H2B.1, H2B/l, H2BFL
HIST1H2BD	209911_x_at	0.97 (0.81-1.17)	0.77	1.01 (0.91-1.12)	0.86	0.92 (0.79-1.08)	0.31	HIST1H2BD, H2B/b, H2BFB
	222067_x_at	1.04 (0.86-1.25)	0.71	1.02 (0.93-1.13)	0.64	0.91 (0.78-1.07)	0.25	H2B/b, H2BFB, HIST1H2BD
HIST1H2BE	208527_x_at	1.16 (0.96-1.4)	0.12	**1.14 (1.03-1.27)**	**0.0087**	**1.18 (1.01-1.38)**	**0.037**	HIST1H2BE, H2B.h, H2B/h, H2BFH
HIST1H2BF	208490_x_at	1.09 (0.91-1.32)	0.36	**1.15 (1.04-1.27)**	**0.0063**	1.11 (0.95-1.3)	0.18	HIST1H2BF, H2BFG, H2B/g
HIST1H2BG	215779_s_at	1.15 (0.95-1.38)	0.16	**1.2 (1.08-1.33)**	**0.00042**	**1.18 (1.01-1.38)**	**0.032**	HIST1H2BG, H2B.1A, H2B/a, H2BFA
	210387_at	1.07 (0.88-1.29)	0.5	**1.27 (1.15-1.4)**	**4.1e-06**	1.1 (0.94.1.28)	0.23	HIST1H2BG, H2B.1A, H2B/a, H2BFA
HIST1H2BH	208546_x_at	1.04 (0.86-1.26)	0.66	**1.14 (1.03-1.26)**	**0.011**	1.09 (0.93-1.27)	0.28	HIST1H2BH, H2BFJ, H2B/j

Table S2. Prognostic data from Kaplan-Meier PLOTTER of Histone genes expression in BC.

**HIST1H2BI**	208523_x_at	1.12 (0.93-1.35)	0.24	1.08 (0.97-1.19)	0.15	1.06 (0.91-1.24)	0.44	HIST1H2BI, H2BFK, H2B/k
**HIST1H2BJ**	214502_at	1.14 (0.94-1.37)	0.18	**1.24 (1.12-1.38)**	**2.3e-05**	1.14 (0.97-1.33)	0.1	HIST1H2BJ, H2BFR, H2B/r
**HIST1H2BK**	209806_at	**1.35 (1.12-1.63)**	**0.001**	**1.27 (1.15-1.41)**	**2.6e-06**	**1.2 (1.03-1.41)**	**0.018**	HIST1H2BK, H2BFT, H2BFAiii
	208579_x_at	1.09 (0.9-1.32)	0.37	**1.25 (1.13-1.39)**	**1.3e-05**	1.11 (0.95-1.29)	0.19	H2BFS, HIST1H2BK, H2BFT, H2BFAiii
**HIST1H2BL**	207611_at	0.98 (0.81-1.18)	0.8	**0.9 (0.82-1.0)**	**0.05**	1.09 (0.94-1.28)	0.25	HIST1H2BL, dJ97D16.4, H2B/c, H2BFC
**HIST1H2BM**	208515_at	0.98 (0.81-1.18)	0.8	1 (0.9-1.11)	0.99	1.04 (0.89-1.21)	0.64	HIST1H2BM, dJ160A22.3, H2B/e, H2BFE
**HIST1H2BN**	207226_at	0.94 (0.78-1.13)	0.48	0.92 (0.82-1.02)	0.11	1.1 (0.95-1.29)	0.21	HIST1H2BN, H2BFD, H2B/d
**HIST1H2BO**	214540_at	0.98 (0.81-1.18)	0.82	0.96 (0.87-1.06)	0.45	**0.84 (0.72-0.98)**	**0.03**	HIST1H2BO, H2B.2, H2B/n, H2BFN
**HIST1H3A**	208575_at	1.01 (0.83-1.22)	0.94	1.07 (0.97-1.18)	0.2	1.07 (0.92-1.25)	0.39	HIST1H3A, H3/A, H3FA
**HIST1H3B**	208576_s_at	1.11 (0.92-1.34)	0.28	**1.2 (1.08-1.33)**	**4e-04**	1.01 (0.87-1.18)	0.87	HIST1H3B, H3/l, H3FL
**HIST1H3C**	208577_at	1.12 (0.93-1.36)	0.22	1.01 (0.92-1.12)	0.78	**1.18 (1.01-1.37)**	**0.039**	HIST1H3C, H3.1, H3/c, H3FC
**HIST1H3D/2AD**	214472_at	**1.29 (1.07-1.56)**	**0.008**	**1.15 (1.04-1.27)**	**0.0067**	**1.28 (1.1-1.49)**	**0.0017**	H3FB, HIST1H2AD, HIST1H3D, H2A.3,
	214522_x_at	**1,27 (1.05-1.53)**	**0.013**	1.1 (0.99-1.21)	0.074	1.0 (0.86-1.17)	0.98	HIST1H2AD, HIST1H3D, H2A.3, H2A/g, H2AFG, H3/b, H3FB
**HIST1H3E**	214616_at	0.91 (0.75-1.1)	0.31	**0.77 (0.7-0.86)**	**5.8 e-07**	1.03 (0.88-1.2)	0.72	HIST1H3E, H3.1, H3/d, H3FD
**HIST1H3F**	208506_at	0.97 (0.8-1.17)	0.72	0.94 (0.85-1.03)	0.19	1.12 (0.96-1.31)	0.15	HIST1H3F, H3FI, H3/i
**HIST1H3G**	208496_x_at	0.97 (0.8-1.17)	0.73	1.26 (1.14-1.4)	5.5e-06	1.06 (0.91-1.23)	0.47	HIST1H3G, H3/h, H3FH
**HIST1H3H**	-	-	-	-	-	-	-	-
**HIST1H3I**	214509_at	0.91 (0.76-1.1)	0.33	**0.87 (0.79-0.96)**	**0.0074**	0.99 (0.85-1.16)	0.91	HIST1H3I, H3.f, H3/f, H3FF
**HIST1H3J**	214646_at	0.93 (0.77-1.12)	0.43	**0.88 (0.79-0.97)**	**0.011**	1.09 (0.94-1.28)	0.25	HIST1H3J, H3/j, H3FJ
**HIST1H4A**	208046_at	1.03 (0.85-1.24)	0.77	0.91 (0.82-1.01)	0.076	1.12 (0.96-1.31)	0.15	HIST1H4A, H4FA
**HIST1H4B**	214516_at	1.11 (0.92-1.34)	0.27	0.99 (0.9-1.1)	0.85	**1.41 (1.2 -1.64)**	**1.6e-05**	HIST1H4B, H4/I, H4FI
**HIST1H4C**	205967_at	1.19 (0.98-1.43)	0.075	1.04 (0.94-1.15)	0.44	**1.34 (1.15-1.57)**	**0.00018**	HIST1H4C, dJ221C16.1, H4/g, H4FG
**HIST1H4D**	208076_at	1.09 (0.9-1.32)	0.37	0.92 (0.83-1.02)	0.098	1.15 (0.99-1.34)	0.072	HIST1H4D, H4/b, H4FB
**HIST1H4E**	206951_at	1.04 (0.86-1.25)	0.68	1 (0.91-1.11)	0.96	1.08 (0.92-1.26)	0.34	HIST1H4E, H4/j, H4FJ
**HIST1H4F**	208026_at	0.92 (0.76-1.11)	0.38	**0.88 (0.8-0.98)**	**0.017**	1.14 (0.98-1.33)	0.087	HIST1H4F, H4, H4/c, H4FC
**HIST1H4G**	208551_at	0.93 (0.77-1.12)	0.43	**0.78 (0.7-0.86)**	**9.2e-07**	1.05 (0.9-1.23)	0.53	HIST1H4G, H4/l, H4FL
**HIST1H4H**	208180_s_at	1.18 (0.98-1.42)	0.086	**1.2 (1.09-1.33)**	**0.00036**	**1.25 (1.07-1.46)**	**0.0049**	H4/h, H4FH, HIST1H4H
	208181_at	0,97 (0.8-1-17)	0.74	0.99 (0.9-1.1)	0.85	1.14 (0.98-1.33)	0.097	HIST1H4H, H4/h, H4FH
**HIST1H4I**	-	-	-	-	-	-	-	-
	214463_x_at	1.04 (0.87-1.26)	0.65	0.99 (0.9-1.1)	0.91	**0.81 (0.69-0.94)**	**0.007**	H4/e, H4FE, HIST1H4J, H4F2iv

Table S2. Prognostic data from Kaplan-Meier PLOTTER of Histone genes expression in BC.

HIST1H4J	208580_x_at	1.0 (0.83-1.21)	0.98	1.0 (0.9-1.1)	0.96	**0.77 (0.66-0.89)**	**0.00068**	HIST1H4J, HIST1H4K, H4F0A22.1, H4/d, H4FD2iv, H4/e, H4FE, H4F2iii, dj160A22.1, H4/d, H4FD
HIST1H4K	208580_x_at	1 (0.83-1.21)	0.98	1 (0.9-1.1)	0.96	**0.77 (0.66-0.89)**	**0.00068**	HIST1H4J, HIST1H4K, H4F2iv, H4/e, H4FE, H4F2iii,
HIST1H4L	214562_at	1.08 (0.9-1.31)	0.4	0.95 (0.86-1.06)	0.36	1.04 (0.89-1.21)	0.65	HIST1H4L, H4/k, H4.k, H4FK
HIST2H2AA3	218279_s_at	**1.22 (1.01-1.48)**	**0.036**	1.08 (0.97-1.19)	0.15	0.9 (0.77-1.05)	0.16	HIST2H2AA3, H2A/q, HIST2H2AA, H2A.2, H2AFO
HIST2H2AA3/4	214290_s_at	1.01 (0.84-1.22)	0.91	1.04 (0.94-1.15)	0.45	0.9 (0.77-1.05)	0.16	HIST2H2AA3, HIST2H2AA4, H2A/q, HIST2H2AA, H2A.2, H2AFO, H2A/r
	218280_x_at	**1.23 (1.02-1.48)**	**0.032**	1.06 (0.96-1.17)	0.26	0.95 (0.82-1.11)	0.54	H2A.2, H2AFO, H2A/r, HIST2H2AA3, HIST2H2AA4, H2A/q, HIST2H2AA
HIST2H2AB	-	-	-	-	-	-	-	-
HIST2H2AC	-	-	-	-	-	-	-	-
HIST2H2BE	202708_s_at	0.93 (0.77-1.13)	0.47	**0.87 (0.79-0.96)**	**0.0073**	0.9 (0.77-1.05)	0.17	HIST2H2BE, H2B.1, H2BFQ, H2B/q
HIST2H3C	-	-	-	-	-	-	-	-
HIST2H4A/B	207046_at	1.02 (0.84-1.23)	0.85	0.98 (0.88-1.08)	0.62	1.15 (0.98-1.34)	0.081	H4F2, H4/o, HIST2H4A,HIST2H4B, H4FN, H4/n, HIST2H4
HIST3H2A	221582_at	1.1 (0.91-1.33)	0.31	1.07 (0.97-1.19)	0.18	1.12 (0.96-1.31)	0.14	HIST3H2A, MGC3165
HIST3H2BB	-	-	-	-	-	-	-	-
HIST3H3	208572_at	1.19 (0.99-1.43)	0.07	1.05 (0.95-1.16)	0.34	1 (0.86-1.17)	0.99	HIST3H3, H3FT, H3/g, H3t
HIST4H4	-	-	-	-	-	-	-	-

For each histone isoform the following data are provided: the affymetrix identifier (affy ID), the HR (hazard ratio, accompanied by the 95% confidence interval in brackets) and the associated p-value for the (i) overall (OS), (ii) relapse-free (RFS), and (iii) distant metastasis-free survival (DMFS). The different target (and synonyms) of the affimetrix probes are shown in the last column. In yellow are evidenced histones with a significative HR > 1, in green those with a significative HR < 1. A HR > 1 implies that patients expressing higher levels of that gene had a worst OS, RFS, or DMFS, the opposite for a HR < 1, i.e. those patients had a better prognosis
